# Anesthesia students’ perception of the educational environment and academic achievement at Debre Tabor University and University of Gondar, Ethiopia: a comparative cross-sectional study

**DOI:** 10.1186/s12909-022-03611-4

**Published:** 2022-07-15

**Authors:** Tadese Tamire Negash, Million Tesfaye Eshete, Getu Ataro Hanago

**Affiliations:** 1 Department of Anesthesia, College of Health Sciences, Debre Tabor University, Debre Tabor, Ethiopia; 2 Department of Anesthesiology and Critical Care, Institute of Health, Jimma University, Jimma, Ethiopia; 3 Department of Anesthesia, College of Health Sciences, Hawassa University, Hawassa, Ethiopia

**Keywords:** Academic achievement, Anesthesia students, Educational environment, Students’ perception

## Abstract

**Background:**

Excellence in Anesthesia education has been advocated to meet the current and future needs of the society. Educational environment plays significant role in determining students’ learning and academic achievement. This study assessed the perception of Anesthesia students on their educational environment and it association with their academic achievement at Debre Tabor University and University of Gondar.

**Methods:**

A comparative cross-sectional study design was employed on 3^rd^ and 4^th^ year students. Dundee Ready Educational Environment Measure (DREEM) was used to assess students’ perception on their educational environment. Perceived performance, Cumulative Grade Point Average (CGPA) and 100 MCQ items were used to assess the academic achievement of the students. An independent t-test was used to assess the differences in the perception of educational environment and academic achievements. Bivariate and multivariable linear regressions were fitted to assess the relationship between perception on educational environment and academic achievement. A *P*-value of less than 0.05 was used to declare the statistical significance.

**Result:**

A total of 123 students (91 from University of Gondar and 32 from Debre Tabor University) were participated on this study. The study showed no statistically significant differences between the groups on the perception of students on the educational environment on DREEM total and subscale scores, and academic achievements. However, there were statistically significant differences in some items of the educational environment measures. On multivariable linear regression; entrance exam result, students’ perception of teachers, students’ academic self –perception and students’ social self-perception showed positive correlation with students’ academic achievement (ß = 0.003 & *P* = 0.04, ß = 0.009 & *P* = 0.9, ß = 0.06 & *P* = 0.42, ß = 0.06 & *P* = 0.39, ß = 0.14 & *P* = 0.015 and ß = 0.13 & *P* = 0.023) respectively.

**Conclusion:**

The perception of students on the educational environment was found to be more positive and there was no statistically significant differences in total and domains of DREEM scores and academic achievement of students between the two institutions. Entrance exam result and more positive perception of the educational environment were positively associated with academic achievement of students.

**Supplementary Information:**

The online version contains supplementary material available at 10.1186/s12909-022-03611-4.

## Background

Educational environment is the diverse physical locations, contexts and cultures in which students learn [1]. It is not only limited to the environment of the classroom, wards, libraries or the medical school as a whole, but also includes the teacher-student relationship, co-operation among classmates, attitude of senior students and staffs. It acts as a hidden curriculum with a major impact on student's learning and academic achievement [2, 3].

The effect of educational environment on the students’ learning and performance can be framed with the social cognitive learning theory which explained as a model of causation involving triadic reciprocal determinism of learning. In this model of reciprocal causation, behavior, cognition and other personal factors, and environmental influences all operate as interacting determinants that influence each other [4]. Moreover, literatures showed that positive perception of students on the educational environment plays significant role in determining students’ learning and academic achievement [3, 5–7]. On the other hand, poor perception of students on their educational environment/perceived mistreatment could have negative consequence on empathy for learning and mental health of students. Studies reported that mistreated students can develop unpleasant feelings, burnout and decreased performance [8–10]. Therefore, students’ ability to develop knowledge, skills and attitude can be linked to their perception with their educational environment, thus placing emphasis on evaluation of the educational environment could have valuable contribution in improving academic achievement of students and producing competent anesthesia professionals.

Due to globalization, the internet and other digital devices are currently the main sources of knowledge and information, the role of higher education institutions should focus on providing higher order learning rather than subject knowledge. It is more valuable for the students to reflect, criticize, analyze and solve problems, instead of reciting what the teacher and the book have stated [11]. The teachers’ role is to be a facilitator, mediator, listener, interpreter, and critical friend to the learners [12].

The government of Ethiopia is making significant efforts in increasing the number of higher education institutions that provide anesthesia programs to increase the number and quality of anesthesia work force in the country. The ministry of health in collaboration with different stake holders has given due emphasis to revise the curricula of undergraduate health programs with the aim of fostering quality of education in health professionals’ trainings. Currently, competency based innovative curricula are being advocated by health science education experts. The essential features of the contemporary curricula are student centered, problem solving, community-based education, integration and early clinical exposure. Whereas the older curricula were more of lecture based, information gathering, hospital based, apparent ship and course based instead of integration. Therefore, there is a paradigm shift in curriculum design from a teacher centered to the student centered approach of learning and teaching which leads to learning how to learn, learning by doing and life-long learning skills [13].

In Ethiopia, there is limited evidence on students’ perception of the educational environment and its effect on the academic achievement and success in anesthesia programs. On top of that, the training of anesthesia programs at higher educations is relatively young in Ethiopia. Furthermore, different anesthesia schools are using different curriculum which could have perceived or actual effect on students’ perception, learning and academic achievement. Therefore, this study assessed the perception of anesthesia students on the educational environment and academic achievement of two universities in Ethiopia, and the relationship of anesthesia students’ characteristics and perception of the educational environment with their academic achievement.

## Methods

### Study area

This study was conducted at Debre Tabor University and University of Gondar, North central of Ethiopia. Debre Tabor University and University of Gondar are among the 23 universities currently providing undergraduate anesthesia program.

Debre Tabor University (DTU) is a young (3^rd^ generation) university which was established in 2008. DTU started College of Health Sciences (CHS) in 2013 with three programs (Medicine, Anesthesia and Midwifery). In contrast to the national harmonized curriculum being implemented in other institutions, the college executes a competency-based hybrid innovative curriculum built on the strengths of the traditional curriculum by incorporating innovative and transformative features highlighted in the SPICES model (student-centered, problem-based, integrated, community-based and systematic). Currently, a total of 67 students are attending undergraduate anesthesia program from 1^st^ to 4^th^ year.

University Gondar (UOG) on the other hand is one of the first generation university which was established in 1954 as a Public Health College and Training Center. The University has steadily growing and evolved into one of the top higher education institutions in the country today. The College of Medicine and Health Sciences (CMHS) is one of the colleges in UOG which uses the conventional curriculum like other old institutions in Ethiopia [14]. Department of Anesthesia is under the college of Medicine which is running both under graduate and postgraduate programs. About 140 Anesthesia students are attending undergraduate program with the conventional curriculum which was revised in 2014.

### Study design and population

Institutional based cross—sectional study was employed on 3^rd^ and 4^th^ year undergraduate anesthesia students.

### Eligibility criteria

All undergraduate anesthesia students who were attending 3^rd^ and 4^th^ year at DTU & UOG in the 2020/2021 academic year were included and students who repeated the same class were excluded from the study.

### Sample size and sampling technique

The sample size was determined using G*power 3.0.10 software using mean difference of two independent (ratio1:3) groups for two tailed students’ t-test, considering standard effect size of 0.8 (large) at marginal error of 0.05 and power of 80%. Based on this assumption the sample size was found to be 17 for group 1 and 51 for group 2 which gives a total of 68. However, during our study period, the total eligible study participants were 32 and 91 students at DTU and UOG respectively. Hence to increase the power of the study, we decided to enroll all 3^rd^ and 4^th^ anesthesia students at both institutions in the study period. So, a total of 123 students were participated on this study. In this study, we selected 3^rd^ and 4^th^ year students due to the suitability of the data collection tools for these batches. Fortunately the data were collected during active class time. As a result all the eligible students were available and volunteered to participate on this study.

### Study variables

#### Dependent variables

The dependent variables of this study were students’ perception of their educational environment as measured with DREEM tool and academic achievement which was measured with perceived performance, CGPA and MCQ exam.

#### Independent variable

Socio-demographic variables like student’s age, gender, religion, region, residency (rural or urban) and income, and institution related variables includes; entrance exam result of grade 12^th^, choice of department and institution/study site are considered as independent variables.

### Data collection process

Data were collected with face-to-face approach using self-administered questionnaire technique at both institutions on the same date and time. The personal and demographic information questionnaire consisted of age, gender, religion, hometown, income, entrance exam result and choice of department. The English version of DREEM questionnaire was adapted and used to assess students’ perception on the educational environment [15]. The questionnaire is comprised of five domains: Students' Perceptions of Learning (SPL); Students' Perception of Teachers (SPT); Students' Academic Self-perceptions (SASP); Students' Perceptions of Atmosphere (SPA); and Students’ Social Self-perception (SSP). Each participant has to read each statement and respond to it using 5- point Likert-type scale which is scored as 4 for strongly agree (SA), 3 for agree (A), 2 for uncertain (U), 1 for disagree (D) and 0 for strongly disagree (SD). We customized/modified some of the statements in the DREEM tool to enable to measure anesthesia learning environment and discussed with five senior anesthetists and pretested the adapted tool.

Academic achievement of the students was measured by using Cumulative Grade Point Average (CGPA) of students were obtained from the registrar of respective universities anonymously, perceived performance on five point Likert scale and 100 MCQ written exam which was administered for 4^th^ year students at both institutions on the same day and time. The 3^rd^ year students were excluded from the MCQ exam because of the difference in curriculum limit us to assess both group of students with the same questions..

All of the data collection tools were prepared in English because the study participants were from all ethnic groups in Ethiopia with diversified mother tongue (language) which makes translation very difficult. To enhance students’ understanding of the tool, we advised them to use dictionary and/or ask data collectors for unclear terms/statements during data collection time.

### Operational definitions

Perception of the educational environment is how students perceive the climate of an institution and the education process. Dundee Ready Education Environment Measure (DREEM) was used to measure students’ perception of the educational environment. The DREEM is an internationally validated 50-statement closed question questionnaire. These 50 items fall into one of the following five subscales: Students’ perception of learning (12 items); Students’ perceptions of teachers (11 items); Students’ academic self-perceptions (8 items); Students’ perceptions of atmosphere (12 items) and Students’ social self-perceptions (7 items). Each of the 50 statements is scored on a five-point scale, with the following labels: ‘‘strongly agree’’ (4), ‘‘Agree’’ (3), ‘‘Unsure’’ (2), ‘‘Disagree’’ (1) and ‘‘strongly disagree’’ (0). Reverse coding was used for items 4, 8, 9, 17, 25, 35, 39, 48 and 50. Thus, higher scores indicate a more positive evaluation. The DREEM has a maximum score of 200, representing an ideal educational environment [5, 7, 16–18]. Individual items of the DREEM scores were classified as **positive** if the mean value is greater than 2 and **negative** if mean value is 2 or below [5, 16]. Subscales of DREEM scores were described using mean values. DREEM overall scores were interpreted as follows: a score of 0–50 as ‘very poor’, 51–100 as indicating ‘plenty of problems’, 101–150 as being ‘more positive than negative’ and 151–200 as ‘excellent’ [1, 7]. Mean scores were used to compare and correlate with learning approaches and academic achievement of Anesthesia students.

Academic achievement was determined based on students’ performance on 100 MCQ items, perceived performance on five point Likert scale and CGPA which were described by mean values [5]. Mean scores were used to compare and correlate with academic achievement.

### Data analysis

Data with complete information were entered into Epi data version 4.20 and exported to SPSS (v20) computer software for analysis. Distributions of variables were checked for normality using histograms, skewness, outliers, Shapiro–Wilk test and Levine’s equality of variances tests. Frequencies, cross tabulations, independent sample t-tests, bivariate and multivariable linear regression were computed and reported using tables and narratives. Mean ± standard deviation (SD) of DREEM scores and academic achievements were used to compare between the groups. The relationship of socio-demographic characteristics and perception on the educational environment with academic achievement were computed using bivariate and multivariable linear regression. A *p*-value of < 0.05 at 95% confidence interval was used to declare statistical significance.

### Data quality management

To ensure the quality of data, a training was given for the data collectors and supervisors. Pretest was conducted on Anesthesia students other than the study sites. The reliability of data collection tool (DREEM) checked with Cronbach’s Alpha = 0.94, mean inter-item = 0.24 and mean intra class correlation coefficient = 0.94 with CI ((0.92 – 0.95); *p* < 0.001)). MCQ items were developed by experienced anesthesia lecturers and reviewed by other experienced lecturers working other than the study sites. To improve the validity and reliability of the MCQ items, we used the nationally prepared assessment blueprint for BSc degree in Anesthesia professional courses developed in 2017. Data were collected on the same day and time at both institutions. Collected data were checked for completeness, clarity, consistency and accuracy by the supervisors and entered to Epi data v4.20.

### Ethical considerations

Ethical approval was obtained from Jimma University Institute of Health Sciences ethical review committee. Then permission letter was submitted to the departments of anesthesia at Debre Tabor University and University of Gondar. The purpose and importance of the study was explained to the participants and written informed consent was obtained from each study participant before data collection. Participants were informed that as there would be no positive or negative rewards for participating or not participating on the study. Data were collected anonymously to ensure the confidentiality of participants’ information.

## Results

### Socio-demographic characteristics of the study participants

All 3^rd^ & 4^th^ year anesthesia students (32 from DTU and 91 from UOG) were participated in this study with 100% response rate. There was no statistically significant difference in socio-demographic characteristics of the study participants (Table 1). Chi-square tests were used to compare the categorical variables whereas mean and standard deviation were used for the continuous variables in the following table (Table 1).

**Table 1:** Socio-demographic characteristics of the study participants at Debre Tabor University and University of Gondar, 2021

Variables	Group		*P*-value
	DTU(*N *=32)	UOG (*N *= 91)	
Age (mean ± SD)	22.06 ±1.19	22.31 ±1.31	.35
Gender (%)			.81
Male	71.9	74.7	
Female	28.1	25.3	
Entrance result (mean ± SD)	485.66 ±15.63	504.84 ±18.07	0.12
Choice of dept (%)			.44
1^st^ choice	96.9	91.2	
Not 1^st^choice	3.1	8.8	
Year (%)			.30
3^rd^ year	59.4	47.3	
4^th^ year	40.6	52.7	
Residence (%)			.20
Urban	28.1	41.8	
Rural	71.9	58.2	
Region (%)			.11
Amara	100.0	91.2	
Others	0.0	8.8	
Religion (%)			.32
Orthodox	100.0	94.5	
Other	0.0	5.5	
Income (mean ± SD)	379.69 ±516.16	417.58 ± 306.9	.62

*SD* Standard Deviation, *DTU* Debre Tabor University, *UOG* University of Gondar, *N* number, *%* percent

### Students’ perception on the educational environment at DREEM subscales

The perception of anesthesia students on the educational environment is classified into five subscales of DREEM scores. The five subscales of DREEM scores includes: 1) Students’ Perception of Learning (SPL), 2) Students’ Perception of Teachers (SPT), 3) Students’ Academic Self—Perception (SASP), 4) Students’ Perception of Atmosphere (SPA) and 5) Students’ Social Self—Perception (SSP). The value of total DREEM and subscale scores are approximately normally distributed and there are no significant outliers.

In this study, there was no statistically significant differences between the groups on the perception of anesthesia students towards their educational environment in all DREEM subscale and total mean score (Table 2).

**Table 2:** Comparison of anesthesia students’ perception on the educational environment at Debre Tabor University and University of Gondar, 2021

DREEM Subscales	Group	N	Mean ± SD	*P*-value
Students’ Perception of Teaching/Learning (SPL)	UOG	91	34.36 ± 8.4	0.74
	DTU	32	33.8 ± 7.4	
Students’ Perception of Teachers (SPT)	UOG	91	30.36 ± 7.75	0.19
	DTU	32	32.34 ± 6.46	
Students’ Academic Self Perception (SASP)	UOG	91	22.5 ± 4.9	0.91
	DTU	32	22.4 ± 3.95	
Students’ Perception of Atmosphere (SPA)	UOG	91	21.56 ± 7.43	0.48
	DTU	32	22.59 ± 5.98	
Students’ Social Self Perception (SSSP)	UOG	91	26.78 ± 6.4	0.25
	DTU	32	28.28 ± 6.26	
Total DREEM Score	UOG DTU	91 32	134.58 ± 27.49 139.46 ± 25.47	0.38

**Key:** *SPL* Students’ Perception of Learning, *SPT* Students’ Perception of Teachers, *SASP* Students’ Academic Self Perception, *SPA* Students’ Perception of Atmosphere, *SSSP* Students’ Social Self-Perception, *SD* Standard Deviation

### Students’ perception on the educational environment at individual DREEM items

#### Students’ perception of teaching

On the individual item analysis regarding students’ perception of the teaching, only students’ perception on the adequacy of clinical exposure showed statistically significant difference between the groups (*p* = 0.007) in which students at UOG had more positive perception than students at DTU (3.3 vs 2.63) with effect size of 0.61 (Table 3).

**Table 3:** Individual DREEM item comparison regarding students’ perception of teaching between Debre Tabor University and University of Gondar, 2021

DREEM items	Group	N	Mean	SD	*P*-value
I am encouraged to participate in the class	UOG	91	3.14	1.02	0.50
	DTU	32	3.28	.95	
I am encouraged to practice in the OR	UOG	91	3.56	.79	0.85
	DTU	32	3.53	.67	
The teaching is sufficiently concerned to develop my confidence	UOG	91	2.81	.96	0.64
	DTU	32	2.72	1.04	
The teaching encourages me to be an active learner	UOG	91	2.76	1.12	0.81
	DTU	32	2.81	1.14	
The teaching is well focused	UOG	91	2.74	1.11	0.82
	DTU	32	2.69	.99	
The teaching is sufficiently concerned to develop my competence (KSA)	UOG	91	2.87	1.07	0.42
	DTU	32	2.69	1.12	
I am clear about the learning objectives of the courses	UOG	91	3.13	.73	0.5
	DTU	32	3.03	.69	
The teaching is often stimulating	UOG	91	2.59	1.07	0.13
	DTU	32	2.91	.81	
The teaching is student centered	UOG	90	2.66	1.08	0.08
	DTU	32	3.03	.93	
Long-term learning is emphasized over short term	UOG	91	2.62	1.17	0.25
	DTU	32	2.34	1.09	
There are adequate skill lab practice sessions	UOG	91	2.21	1.41	0.85
	DTU	32	2.16	1.39	
I am getting adequate exposure for clinical skills expected of me	UOG	91	3.30	.97	0.007*
	DTU	32	2.63	1.21	

*UOG* University of Gondar, *DTU* Debre Tabor University, *N* number, *SD* standard deviation

^*^statistically significant

#### Students’ perception of teachers

Statistically significant differences were observed on the following items: “Teachers are good at providing feedback, teachers get angry at the classroom and/or operation theatre or both, teachers are authoritarian and teachers are patient with their patients” with effect size of 0.49, 0.66 and 0.22 respectively (Table 4) which showed that students from DTU had more positive perception than students from UOG.

**Table 4:** Individual item comparison regarding students’ perception of teachers between Debre Tabor University and University of Gondar anesthesia students, 2021

DREEM item	Group	N	Mean	SD	*P*-value
Teachers are good at providing feedback to students	UOG	91	2.2	1.31	0.001*
	DTU	32	3.0	.91	
Teachers have good communication skills with patients	UOG	91	2.8	1.22	0.06
	DTU	32	3.3	.89	
Teachers are knowledgeable	UOG	91	3.0	1.06	0.06
	DTU	32	3.4	.76	
Teachers give clear examples	UOG	91	2.6	1.21	0.09
	DTU	32	3.0	.71	
Teachers are well prepared for their classes	UOG	91	2.7	1.06	0.05
	DTU	32	3.1	.85	
Teachers provide constructive criticism in clinical practice	UOG	91	2.5	1.24	0.36
	DTU	32	2.7	.94	
Teachers inspire the students	UOG	91	2.3	1.29	0.19
	DTU	32	2.6	1.06	
Teachers get angry in the class and/ OR or both	UOG	90	3.0	1.13	0.04*
	DTU	32	2.4	1.36	
Teachers are authoritarian	UOG	91	2.8	1.10	0.001*
	DTU	32	2.0	1.37	
Teachers are patient with patients	UOG	91	2.8	.97	0.003*
	DTU	32	3.0	.78	
Teachers have adequate skill in clinical skill teaching	UOG	91	3.0	1.02	0.10
	DTU	32	3.4	.83	

*UOG* University of Gondar, *DTU* Debre Tabor University, *N* number, *SD* standard deviation

^*^statistically significant

#### Students’ academic self perception

Regarding students’ academic self-perception, the following items showed statistically significant difference between the groups on three items: “I am able to memorize all I need, much of what I have to learn seems relevant to a career in anesthesia and I am comfortable with the learning strategies being used” with the effect size of *0.54, 0.42* and *0.47* respectively in which students from UOG were demonstrated more positive perception on the first two items. However students from DTU were more comfortable with the learning strategies being used (Table 5).

**Table 5:** Individual DREEM items comparison regarding students’ perception on academic performance between anesthesia students at Debre Tabor University and University of Gondar, 2021

DREEM items	Group	N	Mean	SD	*P*-value
I am able to memorize all I need	UOG	91	2.69	.81	0.01*
	DTU	32	2.25	.80	
Much of what I have to learn seems relevant to a career in anesthesia	UOG	91	3.23	.79	0.03*
	DTU	32	2.88	.87	
I feel I am being well prepared for my profession	UOG	91	3.03	.84	0.14
	DTU	32	2.78	.75	
Last year’s work has been a good preparation for this year work	UOG	91	2.87	1.21	0.49
	DTU	32	3.03	.96	
My problem-solving skills are being well developed here	UOG	91	2.79	.94	0.38
	DTU	32	2.63	.83	
I am confident about passing this year	UOG	90	3.09	1.01	0.85
	DTU	32	3.13	.70	
I have learned a lot about empathy in my profession	UOG	91	2.78	.98	0.05
	DTU	32	3.16	.72	
I am comfortable with the learning strategies being used	UOG	91	2.01	1.37	0.01*
	DTU	32	2.59	1.04	

*UOG* University of Gondar, *DTU* Debre Tabor University, *N* number, *SD* standard deviation

^*^statistically significant

#### Students’ perception of atmosphere

On this subscale only one item “The atmosphere is relaxed during seminars and tutorials” showed statistically significant difference between the two groups with the effect size of *0.44* in which students from DTU showed more positive perception than students from UOG (Table 6).

**Table 6:** Individual DREEM item comparison regarding students’ perception of atmosphere between Debre Tabor University and University of Gondar, 2021

DREEM items	Group	N	Mean	SD	*P*-value
The atmosphere is relaxed during lectures	UOG	91	2.18	1.33	0.14
	DTU	32	1.84	.98	
The practice areas are conducive for learning	UOG	91	2.56	1.15	0.06
	DTU	32	2.13	1.15	
I feel able to ask the questions I want	UOG	91	2.55	1.20	0.17
	DTU	32	2.84	.98	
I feel comfortable in class socially	UOG	91	2.53	1.15	0.14
	DTU	32	2.88	1.18	
There are opportunities for me to develop interpersonal skills	UOG	91	2.84	1.12	0.37
	DTU	32	3.03	.93	
The atmosphere is relaxed during seminars and tutorials	UOG	91	1.85	1.26	0.03*
	DTU	32	2.38	1.12	
The enjoyment outweighs the stress of studying anesthesia	UOG	90	1.89	1.24	0.28
	DTU	32	2.16	1.11	
The atmosphere motivates me as a learner	UOG	91	2.21	1.34	0.53
	DTU	32	2.38	1.12	
I am able to concentrate well for my education	UOG	91	2.90	.98	0.73
	DTU	32	2.97	.93	

*UOG* University of Gondar, *DTU* Debre Tabor University, *N* number, *SD* standard deviation

^*^statistically significant

#### Students’ social self perception

On this subscale only one item, “I have good collaboration with theater staff” showed statistically significant difference between the two groups with the effect size of *0.59*. Similarly, students from DTU have showed better perception on the collaboration with the theatre staff (Table 7).

**Table 7:** Individual DREEM item comparison regarding students’ social self-perception between Debre Tabor University and University of Gondar, 2021

DREEM items	Group	N	Mean	SD	*P*-value
I have good friends in this school	UOG	91	3.27	1.03	0.86
	DTU	32	3.31	1.12	
There is a good support system for students who get stressed	UOG	90	1.79	1.28	0.10
	DTU	32	2.22	1.28	
I am enjoying with this program	UOG	91	2.42	1.18	0.32
	DTU	32	2.66	1.15	
I am rarely bored on the courses	UOG	90	2.38	1.20	0.06
	DTU	32	1.94	1.04	
I get support from other practitioners in practice sites	UOG	91	2.57	1.32	0.66
	DTU	32	2.69	1.23	
My accommodation is pleasant	UOG	91	2.76	.89	0.51
	DTU	32	2.88	.79	
My inter-professional communication is smooth	UOG	91	2.98	1.02	0.48
	DTU	32	3.13	.97	
My social life is good	UOG	91	3.02	1.07	0.63
	DTU	32	3.13	1.04	
I have good collaboration with theater staff	UOG	91	2.69	1.06	0.006*
	DTU	32	3.28	.92	
I receive the necessary clinical supervision	UOG	91	2.89	1.05	0.39
	DTU	32	3.06	.75	

*UOG* University of Gondar, *DTU* Debre Tabor University, *N* number, *SD* standard deviation

^*^statistically significant

### Academic achievement

The academic achievement of anesthesia students was assessed using Perceived Performance (PP) on five point Likert scale, 100 MCQ exams (only for 4^th^ year students) and Cumulative Grade Point Average (CGPA). There was homogeneity of variances for these measurements in the two groups as assessed by Levine’s test for equality of variances (*P* = 0.440, 0.813 & 0.144 respectively).

According to this study, there were no statistically significant differences between the groups on the academic achievements (Table 8).

**Table 8:** Comparison of anesthesia students’ academic achievement at Debre Tabor University and University of Gondar, 2021

Academic Achievement	Group	N	Mean ± SD	*P*-Value
**MCQ Result**	UOG	47	51.96 ± 10.8	0.06
	DTU	13	58.46 ± 10.7	
**Perceived Performance**	UOG	91	3.79 ± 0.87	0.14
	DTU	32	4.06 ± 0.91	
**GPA of Participant**	UOG	91	3.23 ± 0.34	0.91
	DTU	32	3.22 ± 0.40	

*N* number, *SD* standard deviation

## Linear regression

We computed simple and multivariable linear regression to predict the relationship between students’ characteristics and perception on educational environment with their academic achievement (CGPA) as follows

- A.** Relationship between students’ characteristics and their perception on the educational environment**

Multiple linear regression revealed that year of study and perceived performance of anesthesia students are strongly associated with overall DREEM score (β = -11.87; *p* = 0.02 & β = 9.68; *p* =  < 0.001) respectively. As we can see from the following table, year of study showed negative association with overall DREEM score (Table 9).

**Table 9:** Relationship between students’ characteristics and their perception on the educational environment at Debre Tabor University and University of Gondar, 2021

Model		Unstandardized Coefficients		Sig	95% Confidence Interval for B		Collinearity Statistics	
		***B***	***Std. Error***		***Lower Bound***	***Upper Bound***	***Tolerance***	***VIF***
1	(Constant)	158.80	90.42	.08	-20.40	338.00		
	Age	-.98	1.98	.62	-4.92	2.94	.76	1.31
	Sex	.37	6.02	.95	-11.55	12.31	.70	1.42
	Region	4.98	10.56	.63	-15.95	25.92	.72	1.38
	Religion	-2.40	13.16	.85	-28.50	23.68	.72	1.38
	Dept choice	-9.44	9.07	.30	-27.42	8.52	.87	1.14
	Entrance exam	-.04	.14	.76	-.33	.24	.61	1.62
	Residency	-2.68	5.26	.61	-13.11	7.75	.74	1.33
	Income	.00	.00	.47	-.00	.01	.85	1.17
	Year of study	-11.87	5.04	.**02**	-21.87	-1.86	.76	1.30
	Study Site	-.36	5.96	.95	-12.17	11.44	.71	1.39
	GPA	3.47	6.52	.59	-9.45	16.40	.90	1.10
	Perceived Performance	9.68	2.61	**.00**	4.50	14.85	.91	1.09

*Max.VIF* 1.62, ß = beta coefficient (unstandardized), *SE* Standard Error, *CI* Confidence Interval

A **Relationship of Socio-demographic characteristics with academic achievement (CGPA) of Anesthesia Students**
  Socio-demographic variables with p-value of ≤ 0.25 on the bivariate regression were selected for multivariable linear regression. On multivariable analysis, students’ entrance exam result showed strong correlation with CGPA of students (ß = 0.003, *p* = 0.04) (Table 10).

**Table 10:** Multivariable linear regression of socio-demographic variables with CGPA of anesthesia students at Debre Tabor University and University of Gondar, 2021

Variables	Unstandardized coefficients		95% CI	*p*-value
	ß	SE		
**Constant**	1.543	0.83	- 0.095 – 3.18	0.065
Entrance Result	0.003	0.002	0.000 – 0.007	0.04*
Religion	0.202	0.162	- 0.118 – 0.522	0.21
Residency	0.077	0.067	- 0.056 – 0.210	0.25
Year of study	0.064	0.066	- 0.067 – 0.2	0.97
Choice of dept	-0.134	0.125	- 0.38 – 0.11	0.29

Referent categories: urban, 3ry year, orthodox, yes (dept. choice)

*ß* unstandardized beta coefficient, *SE* Standard Error, *R*^*2*^ 0.033, *Maximum VIF* 1.16; * statistically significant
B **Relationship of Students’ Perception on Educational Environment with Academic Achievement (GPA)**

Regarding the educational environments, none of the domains of educational environment is showed strong relationship with students’ GPA. However, Students’ Perception of Teachers (SPT), Students Academic Self Perception (SASP) and Students’ Social Self—Perception (SSSP) have showed positive association with students’ GPA whereas the remaining, Students’ Perception of Learning (SPL) and Students’ Perception of Atmosphere (SPA) showed negative association (Table 11).

**Table 11:** The relationship between anesthesia students’ perception of the educational environment with academic achievement (CGPA) at Debre Tabor University and University of Gondar, 2021

**Variables**	**Unstandardized coefficients**		**95% CI**	**Sig**
	ß	SE		*P*-value
**Constant**	2.99	0.18	2.63 – 3.36	0.00
**SPL**	- 0.00	.00	-0.015– 0.00	0.61
**SPT**^a^	0.00	0.00	-0.01 – 0.01	0.60
SASP^a^	0.00	0.00	-0.010 – 0.02	0.37
**SPA**	-0.00	0.00	-0.019 – 0.00	0.39
**SSSP**^a^	0.00	0.00	-0.010 – 0.20	0.45

*SPL* Students’ Perception of Learning, *SPT* Students’ Perception of Teachers, *SASP* Students’ Academic Self Perception, *SPA* Students’ Perception of Atmosphere, *SSSP* Students’ Social Self-perception, *Max. VIF* 2.4, *R*^*2*^ 1.5%, *ß* beta coefficient (un-standardized), *SE* Standard Error, *CI* Confidence Interval

^a^positive association

## Discussion

The study showed that no statistically significant differences between the groups on the perception of anesthesia students of the educational environment in DREEM subscales and total score, on most of the learning approaches and academic achievement measures. However, there are statistically significant differences in some items of educational environment and learning approach measures. On multivariable linear regression entrance exam result, students’ perception of teachers, students’ academic self -perception, students’ social self-perception, perception on the definition of learning and deep approach to learning showed positive association with students’ academic achievement (CGPA) (ß = 0.003 & *P* = 0.04, ß = 0.009 & *P* = 0.9, ß = 0.06 & *P* = 0.42, ß = 0.06 & *P* = 0.39, ß = 0.14 & *P* = 0.015 and ß = 0.13 & *P* = 0.023) respectively.

### Educational environment

Educational environment is an important bases for learning processes of students and preferences of future workplaces. It is considered as an essential factor in determining the success of an effective curriculum and the students’ academic achievements [19]. This study attempted to assess and compare the perception of anesthesia students on the educational environment between two Universities in Ethiopia. The study showed no statistically significant difference on the perception of anesthesia students of the educational environment between the two institutions at the five DREEM subscales. However, statistically significant differences were observed on some of the individual DREEM items. In contrast to our findings, a study conducted in Pakistan showed significant differences in total DREEM score and subscales between different colleges [20]. The difference could be due to small sample size in our study and difference in study programs.

The current study revealed that the perception of anesthesia students on the educational environment in both universities was found to be more of positive than negative on the total, DREEM subscale scores and individual items. The finding of current study is consistent with studies conducted in Iran, Chinese, UK, Sri Lanka, Saudi Arabia, Nepal, Nigeria, Chile, Jamaica, Sweden, Kuwait, Malaysia, Pakistan and Korea in which more positive perception on the educational environment was found [13, 20–22].

On the other hand a study conducted in Nigeria to compare the perception of students on the educational environment in two Nigerian medical schools offering traditional and student-centered curricula, revealed that the older students in the teacher centered environment showed more positive perception of the educational environment than the younger students in the student centered curricula [16, 19, 23]. This difference might be because of the difference in the study participants of the two Nigerian medical schools.

In contrast to the current study, a study conducted in Egypt among nursing students showed poor perception towards their educational environment. Similarly, contrast results to the current findings were also seen in study conducted in a medical college of Riyadh with the overall DREEM score of 89.9. Studies conducted in medical schools of Iran also reported potential problems with the total DREEM score of 99.6, 98 respectively [24–27]. The reason of this difference might be due to the difference in the study participants and expectation of university students in different countries as a result of differences in the socioeconomic status. Students in low-income country may not expect too much from the Universities and they can easily familiarize themselves with their educational environment. Socio economic status not only affects students’ perception on their educational environment, but also affects the academic achievements of students. This could be the reason why students of both universities in our study have almost comparable and more positive perception on the educational environment [28, 29].

Therefore, even though anesthesia students from both universities demonstrated consistent and positive perception on the total and subscale scores of educational environment measures, it does not mean that students have similar perception in all components of the DREEM items. Thus, improving the aspects of educational environment measures with problems may potentially influence the learning approaches of university students and their academic performance positively [30].

In this study, students with better perceived academic performance are found to have more positive perception with their educational environment. This is explained by positive correlation of students’ perception of the educational environment and academic achievement (GPA). This finding was expected because it has been suggested by different literatures that the more students perceive their learning environment as favorable, the more they score better grade. However, increasing year of study was negatively correlated with perception towards educational environment. Inline to our finding, a cross-sectional study conducted in Sudan to evaluate students’ perceptions of the educational environment and to assess any differences in perception related to students’ performance and their year of study revealed that high achievers’ perceptions of the educational environment are significantly better than those of low achievers and a significant difference was observed between students in different years of study [1].

A nationwide survey was conducted in Korea to assess students' perception of the educational environment of medical schools in Korea on 9,096 students. The overall mean and domains scores of the DREEM were differed significantly between educational systems, grades, genders, and academic achievement levels [22]. In contrast to our finding, graduate-level medical students had higher scores than second year students for the DREEM and its five domains than undergraduate medical students. The differences might be due to differences in study population, study design, learning approaches and sample size. However, more studies are required to determine whether students’ perception on educational environment is more influential on the academic achievement or vice versa by controlling other factors in our country.

### Academic achievement

Academic achievement is an intended competency product of students’ learning process throughout an education program [30]. Similar to the perception of the educational environment, there was no statistically significant difference on anesthesia students perceived performance, MCQ result and CGPA. This similarity might be either due to the small sample size in this study may have failed to detect the differences or could be due to similarity in perception of students on the educational environment and learning approaches or both.

The learning process is an interacting system of three sets of variables: the educational environment and student characteristics (**presage**), students’ approach to learning (**process**) and learning outcomes (**product**) described by Biggs’s et al. [31]. This interaction is explained as; firstly, personal and situational factors influence a student to adopt a particular approach to learning which, in turn, mediates or influences the performance (academic achievement); and secondly, that presage factors (e.g. perceptions of the educational environment) can also directly influence learning outcomes (academic achievement) [30–32].

Even though it is not limited to, our research was framed with the Social Cognitive Theory (SCT) which is a branch of cognitive learning theory. Albert Bandura formulated a comprehensive theory of observational learning that he has expanded to encompass acquisition and performance of diverse skills, strategies, and behaviors. The unique feature of SCT is the emphasis on social influence and its emphasis on external and internal social reinforcements [33]. Social cognitive principles have been applied to the learning of cognitive, motor, social, and self-regulation skills, as well as, moral development, education, health, and societal values. Bandura extended his theory to address the ways people seek control over important events of their lives through self-regulation of their thoughts and actions. The basic process involves setting goals, judging anticipated outcomes of actions, evaluating progress towards the goals, and self-regulating thoughts, emotions, and actions [32, 34].

**Fig. 1:**
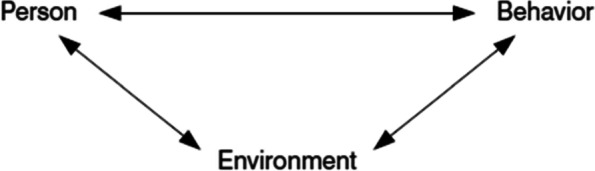
Triadic reciprocal interactive relation between personal characteristics, environmental factors and behavior [35]

Social cognitive theory makes some assumptions about learning and the performance of behaviors. These assumptions address the reciprocal interactions among students’ characteristics, behaviors, and environments (Fig. 1) as; enactive and vicarious learning (i.e., how learning occurs); the distinction between learning and performance; and the role of self-regulation [35].

In this study, we first compared the academic achievements of anesthesia students between the two universities then we tried to investigate the relationship of the perception of students on the educational environment and student characteristics (presage), and their learning approaches with academic achievement (CGPA) of anesthesia students using multivariable linear regression models. We used Cumulative Grade Point Average (CGPA) to correlate academic achievement of anesthesia students with personal characteristics, perception of the educational environment and learning approaches because we believed that CGPA is a better measurement of students’ long-term academic achievement in our study.

In this study university entrance exam result has showed statistically significant positive relationship with their academic achievement (CGPA) (ß = 0.003, *p* = 0.04). Inline to our findings, even though several psychological, cognitive, social and personal factors can affect the academic achievement of anesthesia students, most researchers in education agree that students’ high school performance and entrance exam result are better predictors for their academic performance in the university study [36–39].

From the perception of students on the educational environment; SPL, SASP and SSSP have showed statistically significant positive correlation with students’ CGPA whereas the remaining SPT and SPA showed negative correlation. Regarding the learning approaches of anesthesia students, perception of the students on the definition of learning has showed statistically significant positive relationship with CGPA whereas surface learning approach was negatively correlated with CGPA (ß = -0.17, *p* = 0.023). Anesthesia students’ who prefer using surface learning approaches are high likely to score lower CGPA than those who follow deep approaches of learning. Even though it is not statistically significant, deep approaches to learning showed positive correlation with CGPA. The remaining learning approaches; use of strategic learning approach and preference to different teaching and/or course showed negative association with CGPA.

In this study anesthesia students with more positive perception on the educational environment are more likely to select deep approaches to learning which could have positive correlation with their academic achievement. Inline to our findings, the positive relationship between educational environment, learning approaches and academic performance is supported by studies conducted on different undergraduate health science programs [1, 6, 22, 30, 31, 40]. However, few studies have showed lack of relationship between the perception of educational environment and academic achievement of students [27, 41, 42]. This difference might be due to students’ motivation for learning, non-random sample selection and assessment methods used could have affected the relationship of students’ academic performance and their perception of the educational environment. In addition, students’ habit of study has also significant impact on their academic achievement [43]. However, since it is difficult to address the temporal or causal effect of independent variables with cross-sectional studies, we suggest longitudinal researches to clear this controversy.

### Strengths and limitations of the research

This study is the first study in Ethiopia which tried to compare the perception of anesthesia students towards their educational environment and academic achievement using standardized tools. This study also assessed the relationship between students’ characteristics, perception of the educational environment and academic achievement linking with the social cognitive learning theory.

The limitation of this study is lack of randomization in selection of the institutions and study population which could affect the generalizability of the findings of this study.

## Conclusion and recommendations

In this study, the perception of anesthesia students on their educational environment measures were more positive than negative and there was no statistically significant differences between the two groups of students regarding their perception on the educational environment and academic achievements in general.

On multivariable linear regression, university entrance exam result, students’ perception of teachers, students’ academic self- perception and students’ social self -perception, were positively associated with their academic achievement. Even though, the perception of anesthesia students on the educational environment in both groups was found to be more positive than negative; it was not excellent. Therefore, department of anesthesia at both institutions should work more on improving students’ educational environment for betterment of academic achievement and success. Large-scale longitudinal studies are required to establish the impact of students’ characteristics, educational environment and learning approaches on their academic achievement and success in Ethiopia.

## Supplementary Information

**Additional file 1.** Data collection tool.

## Data Availability

The datasets generated and/or analyzed during the current study are not publicly available due to further analysis is being done from the data sets but are available from the corresponding author on reasonable request.

## Abbreviations

CGPA: Cumulative Grade Point Average
DREEM: Dundee Ready Educational Environment Measure
DTU: Debre Tabor University
EE: Educational Environment
HIC: Hybrid Innovative Curriculum
UOG: University of Gondar
MCQ: Multiple Choice Questions
SPL: Students Perception of Learning
SPT: Students’ Perception of Teachers
SASP: Students Academic Self Perception
SPA: Students Perception of Atmosphere
SSP: Students’ social self-perception
LE: Learning Environment
