# Supplementary material for: Anesthesia students’ perception of the educational environment and academic achievement at Debre Tabor University and University of Gondar, Ethiopia: a comparative cross-sectional study
Source: BMC Med Educ. 2022 Jul 15;22:552. doi: 10.1186/s12909-022-03611-4 (PMC9287941; doi:10.1186/s12909-022-03611-4)
Supplement: Supplementary file 1 — Additional file 1. Data collection tool. [file 12909_2022_3611_MOESM1_ESM.docx]

**Questionnaire**

***Part I: Socio-demographic characteristics of the study participants***

| **Questionnaire** | **Response** | **Remark** |
| --- | --- | --- |
| Age | /____________/year |  |
| Sex | 1. Male 2. Female |  |
| Region | 11 /_________________/ |  |
| r Religion | /__________________/ |  |
| Did you select Anesthesia as your first choice? | 1. Yes 2. No |  |
| W The result of entrance exam? (to be obtained from registrar) | /______________/ |  |
| R Residency (where you come from) | 1. 1. Urban 2. Rural |  |
| Income gained per month (average) | ………. Ethiopian Birr |  |
| CGPA (to be obtained from registrar) | /…………………./ |  |
| Year of study | 1. 2^rd^ 2. 3^rd^ 3. 4^th^ |  |

***Part II: DREEM Questionnaire to assess Students’ Perception of the Educational Environment***

Use: 0 if you strongly Disagree (SD), 1 if you Disagree (D), 2 if you are Uncertain (U), 3 if you Agree (A) and 4 if you strongly Agree (SA) to the following questionnaire and provide your honest response.

***Key****: OR = operation room, KSA = Knowledge, Skill and Attitude*

| Questions/DREEM questionnaire | Response: encircle one | | | | |
| --- | --- | --- | --- | --- | --- |
|  | 0  SD | 1 D | 2 U | 3 A | 4  SA |
| 1. Students’ Perception of Teaching: |  |  |  |  |  |
| 1. I am encouraged to participate in the class | 0 | 1 | 2 | 3 | 4 |
| 1. I am encouraged to practice in the OR | 0 | 1 | 2 | 3 | 4 |
| 1. The teaching is sufficiently concerned to develop my confidence | 0 | 1 | 2 | 3 | 4 |
| 1. The teaching encourages me to be an active learner | 0 | 1 | 2 | 3 | 4 |
| 1. The teaching is well focused | 0 | 1 | 2 | 3 | 4 |
| 1. The teaching is sufficiently concerned to develop my competence (Knowledge, Skill & Attitude) | 0 | 1 | 2 | 3 | 4 |
| 1. I am clear about the learning objectives of the courses | 0 | 1 | 2 | 3 | 4 |
| 1. The teaching is often stimulating | 0 | 1 | 2 | 3 | 4 |
| 1. The teaching is student centered | 0 | 1 | 2 | 3 | 4 |
| 1. Long-term learning is emphasized over short term | 0 | 1 | 2 | 3 | 4 |
| 1. There are adequate skill lab practice sessions | 0 | 1 | 2 | 3 | 4 |
| 1. I am getting adequate exposure for clinical skills expected of me | 0 | 1 | 2 | 3 | 4 |
| II. Students’ Perceptions of Teachers |  |  |  |  |  |
| 1. The teachers are good at providing feedback to students | 0 | 1 | 2 | 3 | 4 |
| 1. The teachers have good communication skills with patients | 0 | 1 | 2 | 3 | 4 |
| 1. The teachers are knowledgeable | 0 | 1 | 2 | 3 | 4 |
| 1. The teachers give clear examples | 0 | 1 | 2 | 3 | 4 |
| 1. The teachers are well prepared for their classes | 0 | 1 | 2 | 3 | 4 |
| 1. The teachers provide constructive criticism in clinical practice | 0 | 1 | 2 | 3 | 4 |
| 1. The teachers inspire the students | 0 | 1 | 2 | 3 | 4 |
| 1. The teachers get angry in the class and/ OR or both | 0 | 1 | 2 | 3 | 4 |
| 1. The teachers are authoritarian | 0 | 1 | 2 | 3 | 4 |
| 1. The teachers are patient with patients | 0 | 1 | 2 | 3 | 4 |
| 1. The teachers have adequate skill in clinical skill teaching | 0 | 1 | 2 | 3 | 4 |
| III. Students’ Academic Self-perceptions |  |  |  |  |  |
| 1. I am able to memorize all I need | 0 | 1 | 2 | 3 | 4 |
| 1. Much of what I have to learn seems relevant to a career in anesthesia | 0 | 1 | 2 | 3 | 4 |
| 1. I feel I am being well prepared for my profession | 0 | 1 | 2 | 3 | 4 |
| 1. Last year’s work has been a good preparation for this year work | 0 | 1 | 2 | 3 | 4 |
| 1. My problem-solving skills are being well developed here | 0 | 1 | 2 | 3 | 4 |
| 1. I am confident about passing this year | 0 | 1 | 2 | 3 | 4 |
| 1. I have learned a lot about empathy in my profession | 0 | 1 | 2 | 3 | 4 |
| 1. I am comfortable with the learning strategies being used | 0 | 1 | 2 | 3 | 4 |
| IV. *Students’ Perceptions of Atmosphere* |  |  |  |  |  |
| 1. The atmosphere is relaxed during lectures | 0 | 1 | 2 | 3 | 4 |
| 1. The practice areas are conducive for learning | 0 | 1 | 2 | 3 | 4 |
| 1. I feel able to ask the questions I want | 0 | 1 | 2 | 3 | 4 |
| 1. I feel comfortable in class socially | 0 | 1 | 2 | 3 | 4 |
| 1. There are opportunities for me to develop interpersonal skills | 0 | 1 | 2 | 3 | 4 |
| 1. The atmosphere is relaxed during seminars and tutorials | 0 | 1 | 2 | 3 | 4 |
| 1. The enjoyment outweighs the stress of studying anesthesia | 0 | 1 | 2 | 3 | 4 |
| 1. The atmosphere motivates me as a learner | 0 | 1 | 2 | 3 | 4 |
| 1. I am able to concentrate well for my education | 0 | 1 | 2 | 3 | 4 |
| V. Student’s Social Self-Perceptions: |  |  |  |  |  |
| 1. I have good friends in this school | 0 | 1 | 2 | 3 | 4 |
| 1. There is a good support system for students who get stressed | 0 | 1 | 2 | 3 | 4 |
| 1. I am enjoying with this program | 0 | 1 | 2 | 3 | 4 |
| 1. I am rarely bored on the courses | 0 | 1 | 2 | 3 | 4 |
| 1. I get support from other practitioners in practice sites | 0 | 1 | 2 | 3 | 4 |
| 1. My accommodation is pleasant | 0 | 1 | 2 | 3 | 4 |
| 1. My inter-professional communication is smooth | 0 | 1 | 2 | 3 | 4 |
| 1. My social life is good | 0 | 1 | 2 | 3 | 4 |
| 1. I have good collaboration with theater staff | 0 | 1 | 2 | 3 | 4 |
| 1. I receive the necessary clinical supervision | 0 | 1 | 2 | 3 | 4 |
